# Interaction of Manganese-Doped Copper Oxide Nano-Platelets with Cells: Biocompatibility and Anticancer Activity Assessment

**DOI:** 10.3390/biomimetics10040203

**Published:** 2025-03-26

**Authors:** Ioan-Ovidiu Pană, Alexandra Ciorîță, Sanda Boca, Simona Guțoiu, Irina Kacso, Maria Olimpia Miclăuș, Oana Grad, Ana Maria Raluca Gherman, Cristian Leostean, Maria Suciu

**Affiliations:** 1 National Institute for Research and Development of Isotopic and Molecular Technologies, 67-103 Donat Street, 400293 Cluj-Napoca, Romania; ovidiu.pana@itim-cj.ro (I.-O.P.); alexandra.ciorita@ubbcluj.ro (A.C.); simona.gutoiu@itim-cj.ro (S.G.); irina.kacso@itim-cj.ro (I.K.); maria.miclaus@itim-cj.ro (M.O.M.); oana.grad@itim-cj.ro (O.G.); raluca.gherman@itim-cj.ro (A.M.R.G.); cristian.leostean@itim-cj.ro (C.L.); maria.suciu@itim-cj.ro (M.S.); 2 Electron Microscopy Center C. Craciun, Faculty of Biology and Geology, Babeș-Bolyai University, 5-7 Clinicilor Street, 400006 Cluj-Napoca, Romania; 3 Interdisciplinary Research Institute in Bio-Nano-Sciences, Babeș-Bolyai University, 42 Treboniu Laurian, 400271 Cluj-Napoca, Romania

**Keywords:** manganese-doped copper oxide nanoparticles, cell interaction, biocompatibility, biomimicry, cancer therapy

## Abstract

Understanding cellular interaction with nanomaterials represents a subject of great interest for the validation of new diagnostic and therapeutic tools. A full characterization of a designed product includes the evaluation of its impact on specific biological systems, including the study of cell behavior as a response to that particular interaction. Copper and copper-based nanoparticles (CuO NPs) have emerged as valuable building blocks for various biomedical applications such as antibacterial and disinfecting agents for infectious diseases, and the evaluation of the metabolism of food, including the iron required for proteins and enzymes or as drug delivery systems in cancer therapy. In this study, the biological impact of manganese-doped crystalline copper oxide (CuO:Mn) nano-platelets on human normal BJ fibroblasts and human A375 skin melanoma was assessed. The particles were synthesized at room temperature via the hydrothermal method. A complete physicochemical characterization of the materials was performed by employing various techniques including X-ray diffraction, electron microscopy, X-Ray photoelectron spectroscopy, and dynamic light scattering. Morphological investigations revealed a flat structure with nearly straight edges, with sizes spanning in the nanometer range. XRD analysis confirmed the formation of the CuO phase with good crystallinity, while XPS provided insights into the Mn doping. The findings indicate that nano-platelets interact with cells actively by mediating essential molecular processes. The exogenous manganese triggers increased MnSOD production in mitochondria, compensating ROS produced by external stress factors (Cu^2+^ ions), and mimics the endogenous SODs production, which compensates internal ROS production as it normally results from cell biochemistry. The effect is differentiated in normal cells compared to malignant cells and deserves investigation.

## 1. Introduction

The development of nanoparticle technology in recent decades has exerted a major impact on diverse scientific fields such as medicine, pharmaceuticals, environmental pollution removal, and energy storage [1,2,3,4,5,6,7]. The ability of nanoparticles to mediate biological effects due to their distinctive physicochemical properties (e.g., increase in an implant’s performance by a nano-patterned surface, enhancement in imaging contrast by superparamagnetic nanoparticles) has been valued through the examination and measurement of the nano–bio interaction. A notable uprising in research has been observed in exploring the biological characteristics of metal nanoparticles, particularly metal oxides, which exhibit promising applications as anticancer, antimicrobial, and antioxidant agents. Cancer, a genetic and metabolic disorder, arises from internal factors such as inherent mutations; immune conditions; and external factors such as infections, alcohol, and tobacco. All these factors influence critical genes’ activity through the formation of cellular intermediates [8,9,10,11], lending to tumorigenesis [12]. Traditional chemo-therapy, while widely used as a form of cancer treatment, comes with a number of drawbacks such as drug resistance and severe side effects [13,14]. As a result, the field gained interest in investigating and developing the chemo-dynamic therapy, a technique based on Fenton-type catalytic reactions that produce highly reactive hydroxyl radicals (OH), which could be further used to induce cancer cell apoptosis while minimizing adverse effects on healthy cells [15,16,17,18,19]. Due to their low side effects and good biocompatibility, manganese nanoparticles present promising potential for clinical applications. Mn^2+^ can be used to initiate the Fenton reaction, which in an acidic environment transforms hydrogen peroxide into more hazardous reactive oxygen species. The catalytic properties of Mn NPs, exhibiting various morphologies, make them good candidates for biomedical applications. The negative charges on the surface of Mn NPs facilitate easy functionalization with a diverse range of biomolecules, achieving dual-purpose application in both diagnosis and treatment. Manganese is generally a non-toxic metal, exhibiting limited adverse effects and primarily causing some degree of nerve damage and reduced exercising capacity when interacting with the brain [20].

Copper oxide (CuO), considered one of the main p-type semiconductors, possesses a face-centered cubic (FCC) crystal structure with copper (Cu) atoms at the corners and oxygen (O) atoms at the center, featuring a band gap ranging from 1.2 eV to 1.9 eV [21,22]. CuO’s combination of semiconducting, magnetic, antibacterial, and optical properties, along with its chemical stability, makes it an outstanding material for a broad array of technological applications. Its potential uses range from energy storage and sensors to biomedical applications and environmental monitoring. As research continues, it is expected that CuO will play an even more significant role in both industrial and medical technologies [23,24]. Copper, while essential in trace amounts for various biological functions, can be toxic to living cells in excessive concentrations. This toxicity stems from its ability to participate in redox reactions, generating reactive oxygen species (ROS) that can damage cellular components [25,26] or, in anaerobic conditions, producing protein aggregation [27]. Additionally, CuO nanoparticles are known for their antibacterial properties, which make them effective against a broad spectrum of microorganisms, including both Gram-positive and Gram-negative bacteria [28,29,30,31]. The release of Cu^2+^ ions from CuO nanoparticles represents the key mechanism by which they exert their antibacterial effect. Cu^2+^ ions disrupt bacterial cell membranes, interfere with cellular functions, and promote the generation of damaging ROS. While copper oxide (CuO) exhibits many beneficial properties, particularly in nanoform, it poses potential toxic risks to both humans and the environment when improperly handled or overexposed. As with any nanomaterial, understanding the dose–response relationship, route of exposure, and size and surface characteristics of CuO is crucial for assessing its toxicity and ensuring safe handling in both industrial and biomedical applications.

In the present work, we inquire regarding the toxicity of copper ions, at low concentrations, which can selectively induce the death of malignant cells while keeping the normal ones unharmed. Specifically, by doping the CuO nanoparticles with manganese (up to 4%), we aim to control the effects of Cu^2+^ release through the simultaneous release of Mn^2+^ ions. The primary objective was to investigate the effects produced by the interaction of CuO NPs and corresponding Cu(II) ions in combination with Mn(II) ions with specific biological environment (herein selected lines of normal human fibroblasts and skin melanoma cells) and to assess the manner by which this nano–bio interaction influences the response of the environment in terms of particle toxicity. To achieve this, the investigated samples of CuO nanoparticles were doped with Mn ions at different concentrations (1%, 3%, and 4%). Structural, compositional, and morphological analyses of the synthesized nanoparticles were conducted using X-ray diffraction (XRD), photoelectron spectroscopy (XPS), and scanning transmission electron microscopy (STEM), respectively. Particle size distribution was determined via dynamic light scattering (DLS). Mitochondrial activity (MTT) and membrane integrity assays were employed to evaluate the toxicological effects. Although CuO nanoparticles and copper-based compounds were previously shown to have potential antitumor effects, their therapeutic use is subject to certain limitations including the promotion of tumor growth, angiogenesis, and occurrence of metastasis by affecting cellular processes [32]. Moreover, it was suggested that the differential response of tumor cells and normal cells to copper-based compounds may depend on the choice of scaffolds and the amount of donor atoms [33,34,35]. Herein, we infer that the presence of Mn at the intracellular level could stimulate the production of manganese superoxide dismutase (MnSOD) at the mitochondrial level and act as a superoxide (O_2_^−^) scavenger, thus indirectly controlling the effect of ROS generation. Thereon, it is expected that these processes will occur differently in malignant cells compared to normal cells. To the best of our knowledge, no study has been reported on the systematic evaluation of CuO nanoparticles’ interaction with the biological environment and the derived biological effects based on the Mn(II) ions doping degree, making it a subject of interest for further investigation and comprehension of its long-term impacts on human health and the environment.

## 2. Materials and Methods

### 2.1. Materials

All reagents involved in the synthesis procedure were used without further purification. Copper (II) chloride dihydrate, 99% purity, was purchased from Alfa Aesar (Kandel, Germany); manganese (II) chloride tetrahydrate, ≥98% purity, was purchased from Sigma-Aldrich (Darmstadt, Germany); potassium hydroxide, 99.4% purity, was purchased from Lach:ner (Továrn, Czech Republic). MTT 3-(4,5-Dimethylthiazol-2-yl)-2,5Diphenyltetrazolium Bromide (Sigma Aldrich, Merck KGaA, Darmstadt, Germany) and LDH (lactate dehydrogenase; a mixture of ionitrotetrazolium violet, phenazine methosulphate, nicotinamide dinucleotide, Li-lactate, and tris) assays (Sigma Aldrich, Merck KGaA, Darmstadt, Germany) were used for cytotoxicity tests.

### 2.2. Copper Oxide (CuO) and Manganese-Doped Copper Oxide (CuO:Mn) Sample Synthesis

Copper oxide (CuO) and manganese-doped copper oxide (CuO:Mn) were prepared by the hydrothermal method, applying the slightly modified steps indicated by A. Khalid et al. [36]. In the first step of the synthesis, aqueous solution (0.5 M) of copper chloride dihydrate was obtained by mechanically stirring at room temperature. The amount of manganese chloride tetrahydrate precursor was determined beforehand in order to obtain a doping percent of 1, 3, and 4 at % and was added to the first solution. The resulting samples were labeled as CuO:Mn1, CuO:Mn3, and CuO:Mn4. A second aqueous solution of potassium hydroxide (1.2 M) was added dropwise over the first solution. The mixture was magnetically stirred for 30 min at room temperature. The second step of the synthesis consists in transferring the formed dark brown precipitate into Teflon autoclave and leaving it for 18 h at 180 °C in the oven. The black particles obtained were washed three times with deionized water and dried in the oven overnight. The detailed synthesis procedure is presented in ref [37].

### 2.3. Characterization Methods

The crystalline structure of the samples was examined by X-ray diffraction using a Rigaku-Smart Lab automated Multipurpose X-ray Diffractometer, which is paired with a high-accuracy θ-θ goniometer and operates in reflection mode with CuKα radiation (λ = 1.54060 Å).

The sample composition, both qualitatively and quantitatively, was determined through X-ray photoelectron spectroscopy (XPS). XPS spectra were acquired using a custom-built SPECS spectrometer equipped with an Mg anode (1253.6 eV) as the X-ray source. For the preparation of powder samples, they were cast onto a sample holder using ethanol. Subsequently, a series of argon (Ar) ion etchings were performed at an acceleration voltage of 1500 V and a filament current of 10 mA, resulting in an Ar^+^ ion current of 2.4 μA. After each etching, the corresponding spectra were recorded. These specific values were selected to prevent any potential artificial reduction in the oxidation states of elements that might occur at higher voltages and currents.

The analysis of the XPS spectra was carried out by normalizing the integral intensities, dividing them by the relative sensitivities, transmission factors, and electronic mean free path factors as provided in the Casa version 2.3.14 software database. Both samples underwent consecutive Ar ion etchings until the shape and intensity of the XPS spectra no longer changed. The analysis of the XPS spectra was conducted using CasaXPS software (version 2.3.14). To calibrate the integral intensities, the raw areas of the spectra were divided by the corresponding relative sensitivity (RST), transmission (T), and electronic mean free path factors (MFP) from the CasaXPS database. The spectra were calibrated with reference to the 284.6 eV values, as the correspondent of the binding energy for adventitious carbon 1 s core-level line. A Shirley background was extracted from the core-level spectra.

Morphological, microstructure, and compositional analyses were carried out on a Hitachi HD2700 scanning transmission electron microscope (STEM), Hitachi High-Tech Corporation, Tokyo, Japan equipped with a cold emission gun and a Dual EDX System coupled with an X-Max N100TLE Silicon Drift Detector (SDD) from Oxford Instruments, Abingdon, UK. The samples were suspended in ethanol, subjected to 10 min of sonication, and subsequently dropped and dried on 400-mesh carbon-coated nickel grinds and plasma-cleaned before imaging under the STEM. All images were collected using an accelerating voltage of 200 kV. Platelet sizes were evaluated by using ImageJ software (version 1.53t) [38] in order to obtain particle size distributions (PSDs).

Particle size distribution, determined via dynamic light scattering (DLS), was measured using the Zetasizer NanoZS90 from Malvern Panalytical Ltd., Malvern, UK. The analysis was performed at a scattering angle of 90° and a temperature of 22 °C. The reported size is based on measured intensity. Size distribution (hydrodynamic diameter—d(nm)) was calculated by considering the value of the Peak1 Mean Intensity (highest % of the Peak Area Intensity). All experiments were performed in triplicate, and the data are expressed as mean ± standard deviation (SD). Samples were ultrasonicated and homogenized before each measurement in ultrapure water (H_2_O-UV) using a test tube shaker (IKA) at a fixed speed of 2800 rpm.

### 2.4. Cytotoxicity Assays

The cytotoxicity assays were performed on normal human fibroblasts (BJ, ATCC CRL-2522, Wesel, Germany) and skin melanoma (A375, ATCC CRL-1619, Wesel, Ger-many) cell types. Cells were grown in DMEM (Dulbecco’s modified EMEM) media supplemented with 10% fetal bovine serum (Gibco, Grand Island, NY, USA), 1% penicillin–streptomycin, and 1% L-glutamine and were kept in a BSL2 (biosafety level 2) incubator at 37 °C with 5% CO_2_ and humidity saturated air. The viability was assessed through MTT, and the membrane integrity was performed on the media using the LDH kit, following established protocols from previous work [39]. In short, cells in exponential phase, at approximately 80% confluency, were trypsinized and counted; then, they were seeded in 96-well plates at 12 × 10^4^ cells/well and were left to attach and reach the exponential growth phase for 24 h. CuO:Mn3 and CuO:Mn4 were selected for cell incubation due to their more uniform distribution of sizes and morphology, ensuring enhanced uptake by the cells. Nanomaterials were suspended in complete medium at 1 mg/mL concentration and kept in the incubator for 24 h prior to the testing. Nanomaterials were diluted to the working concentrations in complete medium and were then dispersed by sonication. Final concentrations of 12.5 to 200 µg/mL were added to cells and left in contact for 24 h. For each concentration, there were 4 duplicates, and 20% Tween 20 was added as a control for cell death. Untreated cells were used as normal viability control.

For MTT assay, a final concentration of 0.5 mg/mL MTT was added to each well and the plates were returned to the incubator for 1.5 h, after which the resulting formazan crystals were solubilized with 2-propanol and the plates were read in a BioTech Epoch ELISA, BioTek, Winoosky, VT, USA spectrophotometer at 540 nm (and 630 nm for background). Mitochondrial activity was calculated based on the untreated control samples’ absorbances, which were considered 100%.

For LDH assay, 50 µL of cell culture medium from each well was transferred to a new plate together with 50 µL of Tris buffer (pH 8), 50 µL of Li lactate, and 50 µL NAD solution, which were added to a final volume of 200 µL per well. This mixture was left to react for 5 min and then was read at the spectrophotometer at 490 nm (and 630 nm for background). LDH release was calculated based on the untreated control samples’ absorbances, which were considered 0%.

Statistical analyses such as One-Way ANOVA, Tukey, and Student’s *t*-test were performed to assess the significance of the results.

## 3. Results

### 3.1. Characterization of CuO and Manganese-Doped CuO Sample

#### 3.1.1. Structural Characterization

**Figure 1:**
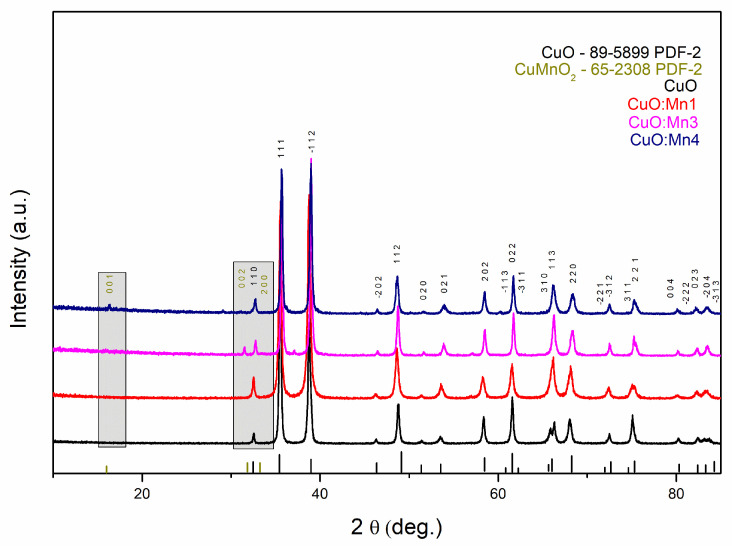
XRD patterns of CuO, CuO:Mn1, CuO:Mn3, and CuO:Mn4.

The crystal structures and phase information of CuO and CuO:Mn were characterized by X-ray diffraction (XRD). The results are presented in Figure 1. The crystalline phases identified in the samples included CuO, corresponding to the PDF-2 database ref. cod 100-089-5899 (space group Cc(9)—monoclinic), and CuMnO_2_, corresponding to the PDF-2 database ref. cod 100-065-5899 (space group C2/m(12)—monoclinic). The most representative diffraction peaks correspond with the crystal planes of copper oxide, specifically (110), (111), (−112), (112), (022), (113), (220), (221), and (−204). Structural changes were observed by XRD, starting from a minimum of 3% Mn doping. Figure 1 illustrates the crystallographic planes for the most relevant diffraction intensities of CuMnO_2_.

The average crystallite size was determined by Halder–Wagner method using PDXP software (16.0), obtaining the following results: 248 Å (CuO), 270 Å (CuO:Mn1), 224 Å (CuO:Mn3), and 384 Å (CuO:Mn4). The differences between nanoparticles are highlighted in Figure 1.

The degree of crystallinity was evaluated as the ratio between the area of diffraction peaks and the total area, which includes both the diffraction peaks and amorphous halos. This analysis employed the MS Reflex Plus module within the Accelrys Materials Studio^®^ suite [40]. No major differences were obtained for the degree of crystallinity of the samples. The obtained values are roughly 90%, an indication of higher crystallinity (see Table 1).

#### 3.1.2. Elemental Composition of the Samples

We conducted a comprehensive X-ray photoelectron spectroscopy (XPS) analysis of CuO:Mn nanoparticles, encompassing both qualitative and quantitative aspects. This analysis involved the examination of core-level lines for Cu *2p*, Mn *2p*, O *1s*, and C *1s*.

When dealing with nanostructured materials, this method, combined with Ar ion etching, allows for precise compositional determinations [41,42]. For this study, our focus was exclusively on the XPS spectra of CuO:Mn3 and CuO:Mn4, as we had previously analyzed a CuO:Mn1 sample in a previous study [37].

**Figure 2:**
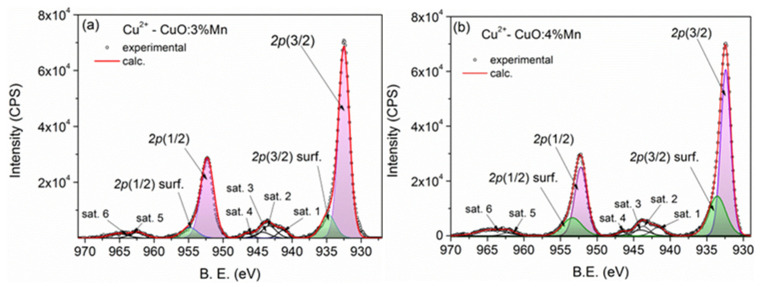
XPS spectra of Cu *2p* core-level corresponding to CuO:Mn3 (**a**) and CuO:Mn4 (**b**). Two Cu positions can be observed, which correspond to the lattice and surface Cu^2+^ ions. The satellite features are specific to the Cu^2+^ state.

The Cu 2p core-level lines of CuO:Mn3 are presented in Figure 2a,b. Within these spectra, a prominent doublet with high intensity is observed, positioned at 932.5 eV for *2p(3/2)* and 952.3 eV for *2p(1/2)*. This doublet corresponds to the lattice Cu^2+^ atoms. Additionally, a less intense doublet, positioned 2.3 eV higher in binding energy, is attributed to surface copper states [37]. The higher binding energies in this doublet result from hydroxide moieties covalently attached to the nanoparticle surface. During the deconvolution process, a spin-orbit splitting (doublet separation) of 19.8 eV was considered, and the peak area ratio was established as A(2p-1/2) = (1/2) A(2p-3/2).

Furthermore, two multiple satellite features are visible at binding energies of approximately 943.7 and 963 eV, which are specific to the Cu^2+^ state. Additionally, a notable Cu LMM Auger line at a kinetic energy of 917.7 eV was observed, indicative of the presence of Cu^2+^. This line had been previously analyzed in previous research [37].

**Figure 3:**
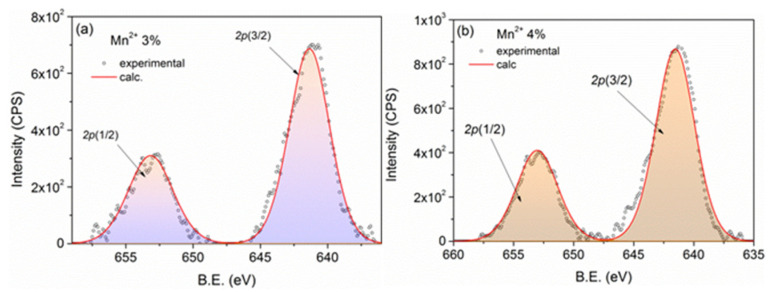
XPS spectra of Mn 2p core-level assigned to Mn^2+^ from CuO:Mn3 (**a**) and CuO:Mn4 (**b**). The corresponding molar doping levels were found to be 2.5% for CuO:Mn3 and 3.5% for CuO:Mn 4.

The core-level spectra of Mn^2+^ *2p* are depicted in Figure 3a,b, exhibiting remarkable similarity. The normalized integral intensities were computed, as previously mentioned. Considering these factors, the estimated Mn doping levels in CuO:Mn3 and CuO:Mn4 are approximately 2.5% and 3.5%, respectively.

**Figure 4:**
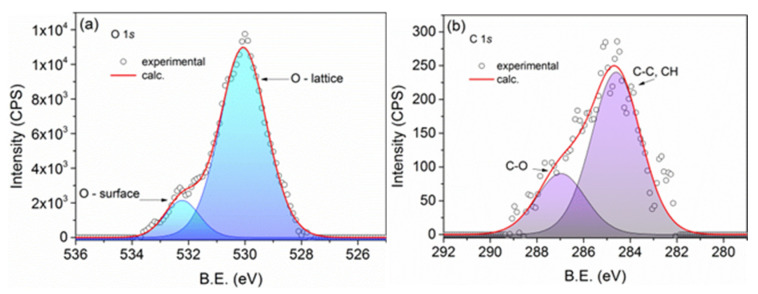
XPS spectra of O *1s* (**a**) and C *1s* (**b**) core-levels recorded from CuO:Mn4. The corresponding spectra for the other samples are similar and are not shown here.

Figure 4a displays the core-level spectra for O *1s*, and Figure 4b presents the spectra for C *1s*, both acquired from CuO:Mn4. Similar spectra were observed in the other samples. In Figure 4a, the high-intensity peak at a lower binding energy corresponds to the presence of oxide, while the higher binding energy peak represents the presence of OH and C-OH groups attached to the surface copper atoms. Notably, there are no C-OOH molecules linked to the nanoparticles, as evidenced by the absence of the corresponding peak in the C *1s* spectrum, shown in Figure 4b. Apart from the residual carbon peak at 286.4 eV, only the C-O peak at approximately 287 eV, associated with C-O bonds, is observed here.

Through quantitative analysis of the XPS spectra, we determined the mass composition of the samples as follows: CuO:Mn1 contains 97.4% CuO:Mn, CuO:Mn3 has 98.9% CuO:Mn, and CuO:Mn4 has a concentration of 98.6% CuO:Mn. The residual carbon, resulting from the chemical preparation method, represents the remaining portion up to 100%. The degrees of molar doping with Mn^2+^ determined for the mentioned samples are 1% (CuO:Mn1), 2.5% (CuO:Mn3), and 3.4% (CuO:Mn4). It was found that the amount of oxygen content in all the samples is lower than the amount of Cu^2+^, indicating the presence of oxygen vacancies in these nanoparticles. In terms of atomic concentration, oxygen vacancies are around 10%.

#### 3.1.3. Morphological Characterization

**Figure 5:**
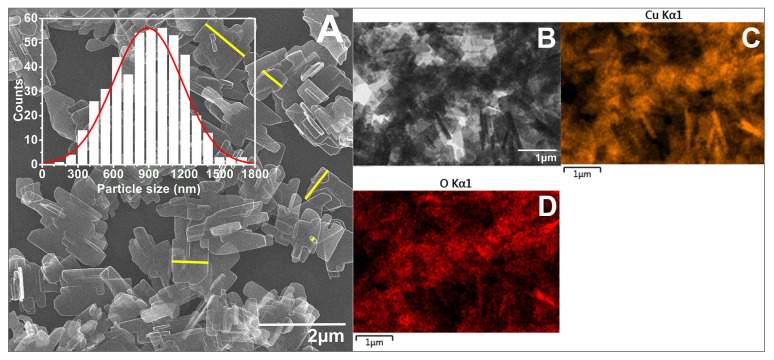
(**A**) SEM and (**B**) TEM micrographs of CuO together with the particle size distribution (number of observables: 462; number of bins: 17) and exemplification of particle size measurement marked in yellow; EDX mapping of (**C**) Cu and (**D**) O elements.

**Figure 6:**
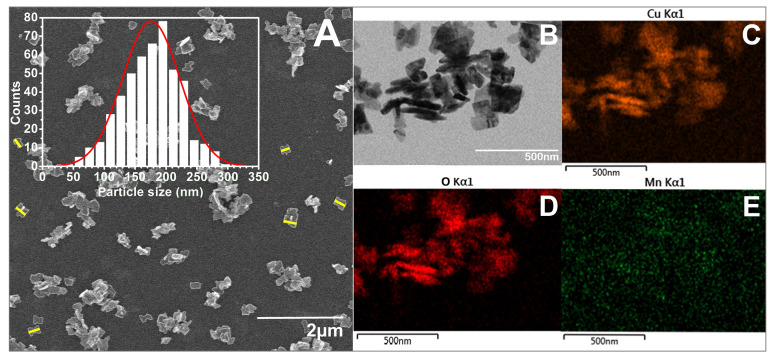
(**A**) SEM and (**B**) TEM micrographs of CuO:Mn1 together with the particle size distribution (number of observables: 482; number of bins: 17) and exemplification of particle size measurement marked in yellow; EDX mapping of (**C**) Cu, (**D**) O, and (**E**) Mn elements.

**Figure 7:**
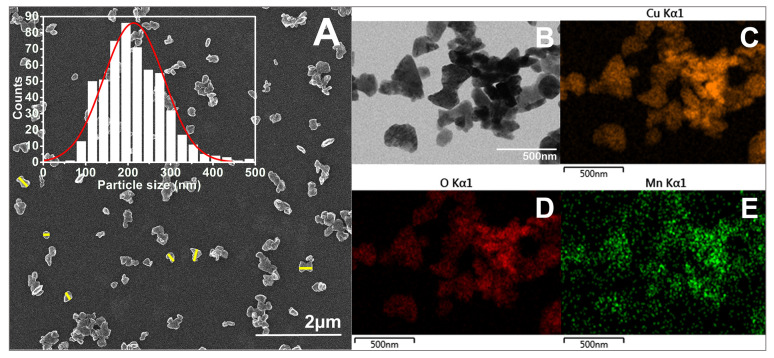
(**A**) SEM and (**B**) TEM micrographs of CuO:Mn3 together with the particle size distribution (number of observables: 534; number of bins: 17) and exemplification of particle size measurement marked in yellow; EDX mapping of (**C**) Cu, (**D**) O, and (**E**) Mn elements.

**Figure 8:**
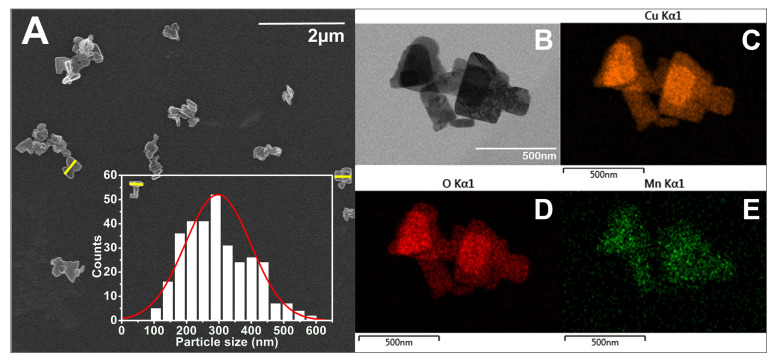
(**A**) SEM and (**B**) TEM micrographs of CuO:Mn4 together with the particle size distribution (number of observables: 316; number of bins: 14) and exemplification of particle size measurement marked in yellow; EDX mapping of (**C**) Cu, (**D**) O, and (**E**) Mn elements.

As seen in the SEM images (Figure 5, Figure 6, Figure 7 and Figure 8), most of the particles exhibit a platelet-like form, characterized by two parallel sides and the other two of irregular shape. Their surface is flat and smooth, without any roughness. When it comes to size, in all cases, the particle size distributions (PSDs) extend over a relatively wide range. The reported sizes correspond to the distance between the two parallel sides of a platelet, or the widest dimension in the case of irregular-shaped platelets. Only those platelets for which the start and end measurement points, as defined above, are not in contact with other particles or platelets having clearly defined margins were considered for measurement. Overlapping platelets or platelet clusters were excluded from the analysis. The selection process was performed manually and is exemplified by yellow markings in Figure 5A, Figure 6A, Figure 7A and Figure 8A. In the case of CuO samples, the microplatelets’ size varies in the range of 154–1700 nm, with an average size of 891 nm. On the other hand, all the other samples registered average sizes in the nanometer ranges: 176 nm (CuO:Mn1), 213 nm (CuO:Mn3), and 297 nm (CuO:Mn4). From the analysis of the distributions of the nanopellets, as shown in the microscopy images, it is found that the size of these nano-objects increases with the increase in the concentration of dopant ions. This increase is especially important in the case of the CuO:Mn4 sample.

Typical sizes of undoped copper oxide (CuO) and manganese-doped crystalline copper oxide (CuO:Mn) samples measured by dynamic light scattering are given in Table 2. As median values, the results corroborate with the SEM measurements and indicate a variation in the average diameter from 120 nm to 392 nm for the Mn-doped samples to 805 nm for the un-doped one, with a polydispersity (PD) index between 0.049 and 0.507 for the most heterogeneous samples. It is worth mentioning that DLS includes measurements of the core particle along with any surface coatings, hydration layers, or adsorbed molecules. Moreover, DLS provides an intensity-weighted size distribution, which tends to emphasize larger particles for which much scattering occurs and often reports a broader size distribution due to aggregates or polydispersity.

#### 3.1.4. Cytotoxicity Tests

Considering the potential application of these materials as antitumor platforms by incorporating specific targeting functionalities, it is imperative to evaluate their cytotoxic effects initially in vitro, using both normal and malignant cells. To this end, two assays were conducted—the mitochondrial activity assay (MTT) and the membrane integrity assay (measured by LDH release)—employing fibroblast cells for normal and melanoma cells for malignant conditions.

**Figure 9:**
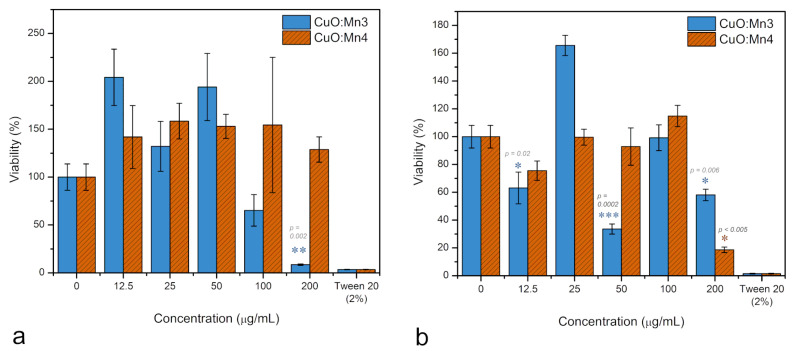
Mitochondrial activity assay (MTT) expressed as % of viability for normal human fibroblast cells (**a**) and human malignant melanoma cells (**b**) treated with the CuO:Mn nanoparticles; the viability was calculated from the mean of at least two independent reactions ± s.e.m. (standard error of the mean); * *p* < 0.05, ** *p* < 0.005, *** *p* < 0.0005, according to Student’s *t* test.

According to the results of One-Way ANOVA and Tukey tests, it was observed that the CuO:Mn3 sample significantly reduced fibroblast mitochondrial activity (*p* < 0.0001) only at high concentrations of 100 and 200 µg/mL (Figure 9a). At these concentrations, the mitochondrial activity values were measured at 65% ± 16% and 8% ± 0.7%, respectively. Conversely, when concentrations between 12.5 and 50 µg/mL were tested within the cytosol of normal fibroblasts, there was an increase of up to 200% in fibroblast mitochondrial activity.

Notably, in contrast with CuO:Mn3, the presence of CuO:Mn4 exhibited an increase in mitochondrial activity across all tested concentrations compared to the control, with values ranging between 140 and 150% (*p* = 0.9, as per ANOVA).

In the examination of melanoma cells (refer to Figure 9b), CuO:Mn3 exhibited oscillating outcomes as a function of dosage, displaying an augmentation in mitochondrial activity of up to 160% ± 0.7% at a concentration of 25 µg/mL, while showing diminished activities of 63% ± 11, 33% ± 3, and 58% ± 4 at concentrations of 12.5, 50, and 200 µg/mL, respectively. Mitochondrial activity is an indicator of metabolic state and, from it, we can infer generalized viability of the cell population exposed to the nanomaterials. If the metabolic activity of cells treated with CuO:MnO3 is very low, that is an indication of nanoparticle toxicity, as the mechanisms that lay behind this result can be cell death, cell stasis, or reduced activity. Some cells, such as cancer cells, may react to a stressful agent by increasing the mitochondrial activity in order to produce more reactive oxidative species to counteract the presence of the xenobiotics. This increased activity usually happens at lower concentrations and this reaction is called hormesis, a nowadays normal reaction to a stressful agent—as an attempt of the cells to adapt to stress. However, this should not be immediately interpreted as increased viability. Conversely, CuO:Mn4 manifested the same oscillatory behavior with smaller amplitude. A notable reduction in mitochondrial activity, specifically to 18% ± 2, is observed at the concentration of 200 µg/mL.

All evaluated nanomaterials displayed variability in MTT absorbance readings (elevated standard deviations), presumably due to the aggregation of the nanomaterials within the cell culture medium. This aggregation resulted in variances in the cellular interactions with the materials. This is more evident for CuO:MnO3, where the polydispersity index values were particularly large (0.507), indicating a high tendency to aggregate. Notably, Tween20 induced a substantial reduction in mitochondrial activity, lowering it to <5%, indicative of cellular demise.

**Figure 10:**
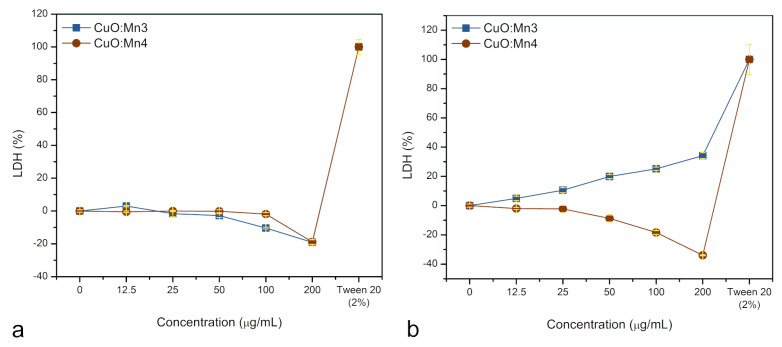
Membrane integrity assay by LDH for normal human fibroblast cells (**a**) and human malignant melanoma cells (**b**) treated with the CuO:Mn nanoparticles; the % of LDH was calculated from the mean of at least two independent reactions ± s.e.m.

In the context of normal fibroblasts, depicted in Figure 10a, LDH release, in case of sample CuO:Mn3, seems to exhibit a slight decline as nanoparticle concentrations increased, resulting in a reduction in LDH to −20% ± 1 compared to the control. For CuO:Mn4 nanoparticles, up to concentrations of 100 μg/mL, the fibroblasts’ cells do not seem to be affected and manage to counteract the cytotoxicity of Cu^2+^ ions. The normal cells seem to react in such a way to preserve the membrane integrity. It should be noted that the presence of interfering nanoparticles affects the assay, causing over-subtraction for the control. A high concentration of nanoparticles inherently interferes with spectrophotometric readings leading to negative results for the blank. This happens if the nanoparticles absorb or scatter light at the wavelength used for tests. It is also possible that negative values are due to cells proliferation in response to the presence of Cu^2+^ ions within the cytosol. Furthermore, at higher concentrations, Cu^2+^ ions may inactivate LDH, leading to a reduction in LDH activity in the treated cells [43]. Additionally, there is a cell proliferation response to the stress induced by excessive ROS production. This response is particularly pronounced in cancer cells. This effect contributes to a false 40% decrease in the readings, reflecting the method’s detection limit.

As illustrated in Figure 10b, LDH release in malignant melanoma cells treated with CuO:Mn demonstrated a Mn concentration-dependent pattern. When CuO:Mn3 is used, a progressively increased LDH enzyme release into the cell culture medium was observed, ranging from a minimum of 4% ± 1 to a maximum of 35% ± 2 with increasing nanomaterial concentrations. Conversely, CuO:Mn4 exhibited an almost dose-independent variation in the LDH release. As mentioned, the negative trend appears probably to cell’s proliferation combined with additional light absorption. What is obvious is that while normal cells remain practically unaffected, the malignant cells’ response to CuO:Mn nanoparticles depends strongly on the doping level of copper oxide with manganese. The mechanism of this process is described in the Section 4.

## 4. Discussion

CuO nanomaterials consistently exhibit significant toxicity towards most living cells. This toxicity is primarily attributed to oxidative stress induced by ROS through their interaction with cell membranes, proteins, mitochondrial function, and nucleic acids. These interactions ultimately result in cell death, through either necrosis or apoptosis [44]. When developing CuO-based materials, it is crucial to consider factors such as concentration, size, crystallinity, surface characteristics, solubility, and doping [45,46,47,48].

When CuO nanocrystals, whether doped or undoped with Mn^2+^, enter the cellular environment, whether intra- or extra-cellular, they release Cu^2+^ ions. This process is followed by the reduction of Cu^2+^ to Cu^+^ ions, typically facilitated by cellular reductants such as glutathione (GSH) or ascorbate, as well as enzymes involved in these metabolic reactions.

For instance,

Cu^2+^ + GSH → Cu^+^ + GSSG (oxidized glutathione) (1)

Cu^+^ + O_2_ → Cu^2+^ + O_2_•^−^ (superoxide anion radical) (2)

Further, reactions of Cu^+^ with hydrogen peroxide (H_2_O_2_), a by-product commonly generated in cells during normal metabolic processes, lead to the production of neutral •OH species:

Cu^+^ + H_2_O_2_ → Cu^2+^ + OH^−^ + •OH (3)

Re-oxidized Cu^2+^ from both Reactions (2) and (3) can be reduced back to Cu^+^, repeating the cycle and generating additional •OH and O_2_• radicals. It is noteworthy that an increased GSSG-to-GSH ratio indicates a greater oxidative stress at the cellular level.

According to Moschini et al. [45], CuO nanoparticles can induce oxidative stress in cells within one hour of interaction, as emphasized by an increase in protein carbonylation, reduced protein-thiol oxidation, reduced viability as determined by mitochondrial activity assay (MTT), and oxidative stress as a result of membrane–nanoparticle interactions. Interestingly, their study found a correlation between the size and crystalline defects of the CuO nanoparticles and the extent of oxidative stress. Wongrakpanich et al. [46] also demonstrated that the larger CuO nanoparticles are, the larger the toxic effect is. Furthermore, they proved that the smaller types of CuO nanoparticles had higher concentrations of ionic Cu in the solution, but this was not as cytotoxic as the presence of large nanoparticles. Moschini et al. [44] also showed that the Cu ions are not the sole determinants of cell death and that the nanoparticles themselves are cytotoxic, at least for human cells in vitro.

For CuO doped with manganese, at low concentrations, a simultaneous release of Cu^2+^ and Mn^2+^ ions is produced within the cytosol. This combination may either enhance or reduce the nanoparticles’ ability to generate ROS within the cellular environment. Thus, at low concentrations, the Mn^2+^ ions may act as controllers of ROS generation. On the one hand, Mn^2+^ ions can participate in redox reactions, affecting the overall redox balance within the cell. Specifically, Mn^2+^ can engage in Fenton-like reactions by cycling between Mn^2+^ and Mn^3+^ states, leading to increased ROS production. The release of Mn ions into the cytosol induces oxidative stress, potentially causing cell death through mechanisms like cell starvation [49,50].

On the other hand, low levels of Mn^2+^ in the cellular environment may enter mitochondria and stimulate the production of manganese superoxide dismutase (MnSOD). MnSOD plays a vital role in protecting cells from oxidative damage by converting harmful superoxide radicals into less toxic molecules such as hydrogen peroxide. Some reports even suggest that MnSOD can promote cell viability and cytoprotection through its antioxidant effects [20,51].

Therefore, depending on the concentration of Mn^2+^ and possibly other factors, its presence may either enhance or mitigate ROS production. As cancer cells often have a higher level of endogenous ROS compared to normal cells, manganese-doped copper oxide nanoparticles can be designed to selectively enhance ROS generation in cancer cells, further disrupting their redox balance and leading to cell death. Normal cells, with their more robust antioxidant systems, are generally less susceptible to this effect. The mechanism of MnSOD scavenging of superoxide and H_2_O_2_ production is described as

$${(Mn}^{2+}-SOD)+O_{2}^{-}\to ({Mn}^{+}-SOD)+O_{2}$$

$$({Mn}^{+}-SOD)+O_{2}^{-}+2H^{+}\to {(Mn}^{2+}-SOD)+H_{2}O_{2}$$

This dual role of Mn has been previously observed, showing different effects on normal and cancerous cells [52]. In our experiments, this could explain the increased mitochondrial activity in both fibroblast and melanoma cells at low concentrations (12.5–50 µg/mL), as well as the general reduction in LDH enzyme activity compared to controls.

Mn doping in the CuO construct induces a protective response in normal fibroblasts, an effect notably discernible at the low tested concentrations of nanomaterials (up to 100–200 µg/mL). The effect is different in malignant melanoma, where the increased Mn doping demonstrates decreased toxicity while lower Mn doping acts in the reversed sense. In all these processes, the Cu^2+^ release is practically the same.

Doping CuO with Mn and inserting these constructs into cellular medium induces a protective response in normal fibroblasts, particularly noticeable at the highest tested concentrations of the nanomaterials (100 and 200 µg/mL). However, in malignant melanoma cells, the effects differ: higher Mn doping levels lead to decreased toxicity, whereas lower Mn doping appears to have the opposite effect. In all cases, the release of Cu^2+^ remains largely unchanged. When Mn is released from CuO:Mn NPs, and it is a question of small amounts, determined by the degree of doping, the production of MnSOD is stimulated, which acts as a superoxide scavenger, thus indirectly controlling the effect of ROS generation due to co-release of Cu^2+^.

The effects and potential biomedical application of CuO:Mn nanocomposites require further testing, yet their potential for chemo-dynamic therapy is obvious. One potential drawback of our synthesized nanocomposites is their broad size range, with averages around 200 nm. This considerable size heterogeneity (emphasized by their PDI values) could pose challenges if administration via intravenous route is envisaged, as they might aggregate within capillaries. However, for more localized or compacted tumors, intra-tumor administration may exploit their larger sizes. The nanoparticles would spread to tumor cells through the enhanced permeability and retention effect [53,54], therefore reducing the need for increased dosage.

From the previously presented mitochondrial activity assay (MTT) performed on normal fibroblasts, for both concentrations of Mn dopant ions, an increase in mitochondrial activity was observed. However, it can be seen how, in the case of the CuO:Mn3 sample, the mitochondrial activity is more intense and more oscillating with respect to the applied dose. In this case, the tendency to compensate for the effect of Cu^2+^ through the increased production of MnSOD is limited by the insufficient concentration of Mn^2+^ ions. When a little greater quantity of Mn^2+^ ions enter the cytosol, as in the case of the CuO:Mn4 sample, the MnSOD production reaches a level sufficient to counteract the effects of ROS generated by Cu^2+^ ions. Overall, normal fibroblasts have a more robust response to the oxidative stress induced by the presence of Cu^2+^ ions, and this seems to be the result of the co-release of Mn^2+^ ions.

In the case of malignant cells, the mitochondrial activity has an oscillating character depending on the CuO:Mn concentration administered. This fact indicates the oxidative stress induced by the administration of Cu^2+^ ions and the tendency to compensate it by using Mn^2+^ ions for MnSOD production is more difficult to equilibrate. This fact is more pronounced in the case of the CuO:Mn3 sample, where the supplied Mn is somewhat smaller and rebalancing is more difficult to achieve.

The membrane integrity assay by LDH sustains these observations. The cell membranes of normal fibroblasts remain almost unaffected at least up to concentrations of 50 μg/mL. For the melanoma cells, low concentrations of Mn^2+^ cannot stop the membrane degradation determined by the Cu^2+^ ions’ oxidative stress; meanwhile, in the case of the CuO:Mn4 sample, the malignant cells seem to protect themselves by MnSOD production through an increased proliferation response. Mechanisms describing the malignant cells’ behavior are not clear and imply various concurrent processes. A possible explanation for this response is that, in the case of the LDH assay, Cu^2+^ ions can induce inactivation through the oxidation of LDH, altering its structure and reducing its ability to catalyze the conversion of lactate to pyruvate. Cu^2+^ can bind to the active site of LDH, blocking its interaction with the substrate. Additionally, Cu^2+^ may react with NADH, a coenzyme involved in the LDH reaction, diminishing its availability for the assay. Cu^2+^ may also interfere with the reagents or detection methods used in the LDH assay, compromising the accuracy of the measurements [55]. These aspects contribute to the negative LDH values at high concentrations of nano-objects in cytosol in case of normal cells. Normal cells resist at high values of both CuO:Mn3 and CuO:Mn4, at least up to 100 μg/mL. Contrarily, in the case of malignant cells, CuO:Mn3 produces significant cell death mostly by necrosis (membrane ruptures and LDH release) while CuO:Mn4 induces a rather apoptotic cells death (lower LDH release).

As previously mentioned, these observations are valid for low doping concentrations of CuO with Mn ions. Of course, the administration of large amounts of CuO:Mn type constructs has a catastrophic effect both in the case of normal fibroblast cells and in the case of malignant cells. The main idea is to find an optimal concentration to maintain the functioning of normal cells and to determine the destruction of malignant cells by oxidative stress.

## 5. Conclusions

In this paper, we present our findings on the biological interaction of copper oxide (CuO) nanoparticles having different levels of manganese dopant, herein 1%, 3%, and 4% with two specific lines of cells and the results that derive from this particular interaction related to the cell behavior. The nanoparticles were successfully synthesized via the hydrothermal method at room temperature. Their biological impact was evaluated by subjecting the particles to normal human BJ fibroblast cells and human A375 skin melanoma cells. The results revealed a correlation between the physicochemical characteristics of the nanoparticles and the biological effects on the tested cells. Moreover, it became evident that the cytotoxicity of the nanoparticles is influenced by their doping degree with manganese. An optimal quantity of Mn^2+^ ions within the cytosol, in the case of normal cells, enhances the production of manganese superoxide dismutase at mitochondrial level, which is a biological process that counterbalances the ROS generation produced by Cu^2+^ ions. This way, the normal cells seem to be more protected than the malignant ones. In the case of the latter, a lower resistance to the presence of Cu^2+^ ions is found, with the MnSOD generation mechanism manifesting itself only at higher concentrations of Mn^2+^ ions. These distinctive characteristics make our designed nanocomposites promising candidates for further and more targeted investigations for therapeutic purposes. The mechanisms and outcomes of ROS generation in living cells can vary depending on factors such as nanoparticle concentration, exposure duration, and the involved cell types. Therefore, it is imperative to carefully control and monitor the effects of such nanomaterials in biomedical settings in order to maximize their benefits. This could be achieved, for example, through the use of nanoplatform assays that can maintain a constant level of Cu ions and variable concentrations of Mn ions in the cytosol. Also, by conjugating these types of nanoplatforms with various biomolecules, fine-tuning of the levels of Cu and Mn ions that pass into the cytosol can be obtained, which is an important characteristic of biomimicry functionality.

## Figures and Tables

**Table 1:** Crystallinity of sample nanoparticles.

Sample	Degree of Crystallinity (%)
CuO	90.44
CuO:Mn1	89.30
CuO:Mn3	88.61
CuO:Mn4	90.73

**Table 2:** Particle size analysis results from DLS.

Sample	Hydrodynamic Diameter (nm)	PDI
CuO	805.26 ± 49.68	0.182
CuO:Mn1	258.73 ± 25.31	0.328
CuO:Mn3	120.94 ± 28.10	0.507
CuO:Mn4	392.66 ± 26.51	0.049

PDI: polydispersity index.

## Data Availability

The original contributions presented in the study are included in the article, further inquiries can be directed to the corresponding author.
